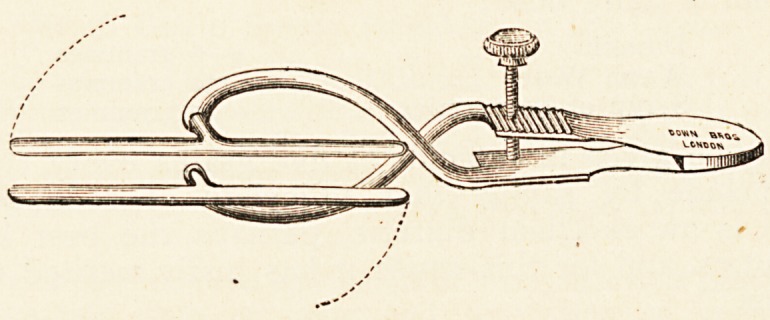# Notes on Preparations for the Sick

**Published:** 1894-06

**Authors:** 


### Notes on Preparations for the Sick.

**Food for Infants and Invalids. Food Biscuits.**—G. Mellin, London. **Emulsion of Cod-Liver Oil and Hypophosphites.**—Mellin's Emulsion Co., Ltd., London.—Mellin's food needs little description. Its chemical composition concurs with the results of experience in indicating that it is a perfect food for infants and persons of weak digestion. Every trace of starch has been converted into grape-sugar or dextrine; it contains 16 per cent. of nitrogenous matter, and only 6 per cent. of insoluble residue.

The **Biscuits** contain about 50 per cent. of the food; they appear to be very suitable for infants during the transitional stage, and for invalids.

The formula of the **Emulsion** is as follows:—

| | | |
|---|---|---|
| ℞ | Ol. Morrhuæ ... ... ... ... ... ... | ℥vi |
| | Gum. Arabic. ... ... ... ... ... ... } | |
| | Sacch. Alb. ... ... ... ... ... ... } | āā q.s. |
| | Aq. Cinnamomi ... ... ... ... | ad. ℥xii. |
| | Misce ut fiat Emuls. et adde: | |
| | Calcii Hypophosph. } | |
| | Sodii Hypophosph. } | āā gr. 48. |

The adult dose is from one to two table-spoonfuls. It should prove a useful preparation, and deserves especial favour as its exact composition is stated.

**Virol.**—Liquor Carnis Co., London—.This is said to be "a highly concentrated 'complete food,' consisting of the proteids of beef and eggs, the fats of beef and eggs, the marrow of beef or essence of bone, the carbohydrate extract of malt,—and the salts of beef and eggs (including the lime-salts of the shell), in proportions carefully adjusted to diet-formulæ laid down by the most up-to-date physiologists." Such is the description of a preparation which is not unpalatable, reminding one of Christmas plum-pudding, and which many patients will take who decline cod-liver oil; but it can in no sense be looked upon as a substitute for oleum jecoris aselli in the treatment of phthisis.

---

**Mist. Calc. Chlor. c̄ Ferro. Mist. Calcis Chlorid. Ol. Morrhuæ Etheris. Lotio Picis Carbonis Co. Linimentum Pinus Aceticum. Lotio Capitis. Lin. Tannici Co. Chloralose — in cachets.** — Arthur & Co., London.—We doubt not but that these are all excellent and useful preparations; but we should like to see the formula printed on the label before we venture to express any opinion upon them.

---

**Wine of Cod-Liver Oil with Peptonate of Iron** (Stearn's). **Cascara Aromatic** (Stearn's). **Dike's Pepsin.**—Thomas Christy & Co., London.—It has long been thought that the active principle of cod-liver oil was the oil itself, but recently the practice of giving certain solid extracts from the oil has come into vogue; and there is much clinical evidence in favour of the utility of such preparations as morrhuol, in which little or none of the oily matters are retained. Various physiological experiments tend to show that cod-liver oil is a reconstituent of the tissues through its richness in phosphates, phospho-glyceric acid, and organically combined phosphorus. Bromine and iodine also contribute; but it is presumed that the principal medicinal value of the oil may be due to such substances as butylamine, amylamine, morrhuine, and morrhuic acid. Presuming that cod-liver oil owes its value as a medicine to the active principles which may be separated from the oil itself, we have good grounds for encouraging the use of capsules containing these separated products, or of fluids which contain them in solution.

The **Wine of Cod-liver Oil** (Stearn) is an elegant fluid, containing 25 per cent. of the extractives from the oil, together with four grains of peptonate of iron to each fluid once. It is pleasant to the taste, does not cause any nausea or eructation; and it has been found to act like cod-liver oil in improving nutrition and increasing the weight. In the large class of patients who object to the crude oil, this wine is well worthy of trial.

**Cascara Sagrada** still maintains its position as one of the most agreeable and efficient laxatives at our disposal. Stearn's aromatic production is a concentrated one, from which the bitter principle has been removed, and which may serve as an excellent vehicle for many other useful drugs. It has given excellent results.

The **Pepsin** has a digestive strength of one in 3000; it is free from odour and taste and is not hygroscopic. It makes a clear solution, which does not precipitate with acetic acid: it is, therefore, free from mucus. It may be dispensed in powder form or combined with other powders. Clearly a pepsine like this in scales, tasteless, odourless, dry, and easily soluble in water, has advantages over many other kinds in daily use.

---

**Beef Jelly. Fluid Beef Jelly. Beef Meal. Beef-Cacao. Bromelin. Esencia de Coca. Fluid Ext. Cocillaña.**—MOSQUERA-JULIA FOOD COMPANY, Detroit.—The dietary of cases of chronic malnutrition becomes daily a more and more important question; and hyper-alimentation in the treatment of such diseases as phthisis is becoming the rule. Meat and milk are the two articles of food which furnish the greatest amount of nutriment with the least expenditure of digestive energy. The latter is frequently declined, and the former cannot be taken excepting in some artificially prepared medicinal form. Of the many preparations of meat which have been devised for the invalid, the Mosquera foods, samples of which have been sent to us by Messrs. Parke, Davis & Co., are the newer candidates for professional favour. They have already given excellent results, and are found to be agreeable to the taste, easy of digestion, readily assimilable, and efficient in improving nutrition.

The **Beef Jelly** contains over 50 per cent. of albuminous matter; and the **Beef Meal** 77.25 per cent. Prof. Chittenden, of Yale College, estimates the nutritive value of the latter to be at least that of six times its weight of lean beef. It is a partially pre-digested powdered beef, containing 18.34 per cent. of peptone, but without the nauseous bitterness which characterises most preparations of peptone. The **Beef-Cacao** has cacao and sugar added, and the flavour of this meat is completely overcome by the more powerful flavour of the cacao. These powders might well take the place of the raw meat, and the meat juices, which are now so universally in demand where the digestive powers are weak and hyper-alimentation is needful. Such powders as these are destined to play an active part in the treatment of phthisis and other disorders of nutrition.

**Bromelin** is the name given to a proteolytic ferment derived from the juice of the pine-apple. It possesses in a high degree the properties of animal pepsine.

The **Esencia de Coca** is an agreeable liqueur, each fluid ounce of which represents 32 grains of the erythroxylon coca.

We have used the **Fluid Ext. Cocillaña** in several cases of acute and chronic bronchitis and in bronchitic asthma. It

11

seemed to be most beneficial in the earlier stages of acute inflammatory conditions of the larger bronchi before expectoration had become free, and in more chronic forms with deficient secretion. It appears to stimulate the mucous glands, and, in fact, to resemble ipecacuanha in its action, rendering the expectoration more copious and less tenacious; but the advantage of this drug over the better known expectorants was not very obvious. We have generally prescribed 20 to 25 minims every two hours till three doses have been taken, and then continued every four to six hours. These doses never gave rise to any nausea, and herein lies its sole recommendation as a substitute for ipecacuanha and apomorphine.

---

**Oil of Eucalyptus. "Lanoline," Pine Tar Soap.**—BURROUGHS, WELLCOME & CO., London.—The **Oil** is a strong preparation obtained by distillation from eucalyptus leaves. It is a valuable antiseptic, which may be used for inhalation better than almost any other substance, on account of its pleasant odour and unirritating properties.

The **Soap** is an excellent antiseptic preparation, and the presence of lanoline makes it penetrative, especially when used for the hair. The odour left after its use is less disagreeable than that of carbolic acid, but it still leaves something to be desired.

---

**Phenosalyl.**—FARBWERKE VORM. Meister LUCIUS & BRUENING, Hoechst-on-the-Maine.—This preparation—another new antiseptic!—invented by Dr. J. de Christmas, of Paris, has been forwarded to us by Messrs. Burroughs and Wellcome, who are the agents for it in the United Kingdom.

Investigations are constantly being made with a view of finding a perfect antiseptic. Corrosive sublimate and phenol have certain objectionable qualities which it is sought to avoid without loss of efficiency.

**Phenosalyl** is a mixture of carbolic, salicylic, and benzoic acids, melted together and dissolved in lactic acid. Its odour is certainly less objectionable and less persistent than that of carbolic acid; while one or two trials which we have been able to give it proved it to be a powerful antiseptic in solutions of the strength of only 1 per cent.

---

**Dr. James Swain's Clamp for Enterectomy.**—DOWN BROS., London.—The chief advantage of this instrument consists in the uniform pressure which exists along the whole length of the blades, whether they are parallel or not. It therefore compensates for any variation in thickness at the mesenteric and free borders of the intestine, and obviates that excessive pressure at the proximal end, and the feeble pressure at the distal end, which obtains in many clamps. This equality of pressure is secured by the swinging of the blades—which are supported at their *centre* by short secondary shanks—on pivots at the end of each main shank. The spring handle of the clamp is after the model of Dieffenbach's forceps, with a screw for increasing the power if necessary. The shanks are long and curved outwards, so as to allow the bowel, when held by the blades, to pass through them without touching. Before use each blade is covered with a piece of rubber drainage-tube. It is made in two sizes, with blades three inches and two inches in length respectively.

---

**Automatic Grip.**—B. HUNTER, Bristol.—This is an ingenious provision for holding an abdominal binder in position after a confinement or an abdominal section. It allows a perfectly controlled support or compression without the use of pins or hooks. We have used it with much success in a case of umbilical hernia, but have been disappointed with it in a case of fractured ribs, for which it is also recommended. Its chief use will doubtless be immediately after a confinement.

---

**Agathin. Diuretin.**—BURROUGHS, WELLCOME & Co., London. —The first of these preparations, the scientific name of which is salicyl-*a*-methyl-phenyl-hydrazone, is said to have analgesic properties in articular rheumatism, neuralgia, and sciatica. It is best given in cachet, in doses of four to eight grains three times daily. We have tried it in a case of severe and advanced locomotor ataxia, but it entirely failed to give relief.

Many observations of numerous observers lead to a uniform conclusion that diuretin is of great value in dropsies of both renal and cardiac origin. Dr. Silvestri reports as follows:— "The salicylate of soda and theobromine in the early stages of asystole has a very limited action, undoubtedly inferior to digitalis, strophanthus, convallaria, etc. It is in the last stages of asystole (œdema and anasarca) that its beneficial effects are more often shown, when the heart-muscle is degenerated. It then acts, as we have seen, by influencing the kidney and exciting its function. Of all the forms of cardiac disease, it acts most energetically in mitral lesions, less in aortic disease, and less still when to one or other is joined diffuse atheroma of the peripheral arterial system. Of the forms of renal affection, it acts most satisfactorily in acute nephritis—somewhat less so in chronic nephritis. The diuretic effects of diuretin become manifest after 12, 14, or 24 hours. The best mode of administration is a solution in warm water. One prescription was as follows for a daily dose:

| | |
|---|---|
| Diuretin ... ... ... ... ... ... | 4—6 grammes |
| Warm Water ... ... ... ... | 130 grammes |
| Syrup of Peppermint ... ... | 20 grammes |

or it may be given in tabloids of five grains every two hours. We conclude by saying that salicylate of soda and theobromine is an excellent diuretic, perhaps the best known to therapeutics, and for this cause it has justly merited the name 'Diuretin.'"

---

**Ophthalmic Tabloids and Holder. Chromatic Dial.** Burroughs & Wellcome, London.—These **Tabloids** are minute discs, prepared with a sterile, innocuous, non-irritating basis; they are more soluble than gelatine discs, and are easily applied to the eyeball by means of the holder, which is made like a syringe, and consists of a small india-rubber ball with a celluloid nozzle, the end of which is just large enough to hold a tabloid. Nine tubes of different kinds of tabloids, with a holder, and a small pestle and mortar are included in a neat case at the moderate price of 7s. 6d.

The **Chromatic Dial** is a convenient test for colour blindness. Accompanying it are directions by which the test is easily applied, and in many cases it would prove of service as a preliminary investigation, which, however to be made complete, should be verified by the use of Holmgren's wools.

---

**Cliftonia.** H. W. Carter and Co., Bristol.—This is an aerated distilled water, and as such is all that can be desired. For purposes associated with conditions where flushing of the tissues and the washing away of excreta are needful, more especially when the excreting organs are hampered by disease, the need for distilled water as a beverage is obvious, and its aeration gives it agreeable qualities which are scarcely surpassed by any of the natural waters such as Apollinaris. The sterilisation of the water by distillation gives a guarantee of freedom from deleterious impurities of all kinds.

---

**Golden Hop Stout.** J. and T. Usher, Bristol.—This semi-medicinal beverage has an appearance resembling that of ordinary stout, but no one could mistake the one for the other. Its flavour is not unpleasant, and it has no exhilarating qualities; but as a daily drink we should soon be weary of it.

## Figures and Tables

**Figure f1:**